# Transcriptional Profiling of Rice Early Response to *Magnaporthe oryzae* Identified OsWRKYs as Important Regulators in Rice Blast Resistance

**DOI:** 10.1371/journal.pone.0059720

**Published:** 2013-03-27

**Authors:** Tong Wei, Bin Ou, Jinbin Li, Yang Zhao, Dongshu Guo, Youyong Zhu, Zhangliang Chen, Hongya Gu, Chengyun Li, Genji Qin, Li-Jia Qu

**Affiliations:** 1 State Key Laboratory for Protein and Plant Gene Research, College of Life Sciences, Peking University, Beijing, China; 2 Agricultural Environment and Resources Research Institute, Yunnan Academy of Agricultural Sciences, Kunming, China; 3 College of Biological Sciences, China Agricultural University, Beijing, China; 4 College of Agronomy and Key Laboratory for Plant Pathology of Yunnan Province, Yunnan Agricultural University, Kunming, China; 5 College of Agriculture and Biotechnology, China Agricultural University, Beijing, China; 6 The National Plant Gene Research Center, Beijing, China; China Agricultural University, China

## Abstract

Rice blast disease is a major threat to rice production worldwide, but the mechanisms underlying rice resistance to the causal agent *Magnaporthe oryzae* remain elusive. Therefore, we carried out a transcriptome study on rice early defense response to *M. oryzae*. We found that the transcriptional profiles of rice compatible and incompatible interactions with *M. oryzae* were mostly similar, with genes regulated more prominently in the incompatible interactions. The functional analysis showed that the genes involved in signaling and secondary metabolism were extensively up-regulated. In particular, *WRKY* transcription factor genes were significantly enriched among the up-regulated genes. Overexpressing one of these *WRKY* genes, *OsWRKY47*, in transgenic rice plants conferred enhanced resistance against rice blast fungus. Our results revealed the sophisticated transcriptional reprogramming of signaling and metabolic pathways during rice early response to *M. oryzae* and demonstrated the critical roles of WRKY transcription factors in rice blast resistance.

## Introduction

Plants are frequently challenged by various kinds of pathogens in their living environments. To survive pathogen attack, plants have evolved two layers of immune systems: pathogen-associated molecular pattern (PAMP)-triggered immunity (PTI) and effector-triggered immunity (ETI)[1]–[3]. PTI is mediated by pattern recognition receptors (PRRs) that recognize PAMPs, whereas ETI is mediated by resistance (R) proteins that recognize pathogen effectors. Generally, PTI and ETI trigger similar defense responses, but ETI is much faster and quantitatively stronger [2]–[4].

As one of the most important staple crops, rice (*Oryza sativa*) feeds about half of the world’s population. However, 10–30% of annual rice production worldwide is lost to rice blast disease, caused by the fungal pathogen *Magnaporthe oryzae* [5], [6]. Because rice blast disease poses a serious and recurrent problem, the rice–*M. oryzae* interaction has been studied for decades. The infection starts with the attachment of fungal spores to plant leaf surfaces. Within 24 h post-inoculation (hpi), fungal spores germinate and form infection-specific appressoria to penetrate leaf cuticles and invade epidermal cells [5], [7]. It has been suggested that 24 hai is a critical point for pathogen invasion [5]. After fungal invasion, the rice–*M. oryzae* interaction obeys Flor’s “gene-for-gene” hypothesis [8]. If fungal effectors are recognized by cognate rice R proteins, ETI is triggered and culminates in hypersensitive response (HR) that halt fungal growth within 48 hours. If fungal effectors are not recognized, a limited PTI response occurs upon the recognition of fungal PAMPs, such as chitin [9], [10], and fungal hyphae continue to spread through plant tissues, leading to disease symptoms after days [5], [11]–[13]. Thus, the early defense response immediately after *M. oryzae* invasion is critical to the final outcome of rice blast resistance.

Genetic approaches have identified defense regulators in rice blast resistance. The rice homologue of *Arabidopsis* Nonexpresser of PR Genes 1 (NPR1), NH1, plays a positive role in rice blast resistance, as knockdown of *NH1* impaired rice blast resistance, whereas overexpression enhanced resistance [14]–[16]. Transgenic plants overexpressing *OsWRKY13*, *OsWRKY31*, *OsWRKY45*, and *OsWRKY53* showed enhanced resistance to *M. oryzae*, indicating their important roles in rice blast resistance [17]–[21]. Large-scale approaches have also been used to study rice early response to *M. oryzae*, including EST sequencing [22], robust-long serial analysis of gene expression [23], proteomics [24], [25], and microarrays [26]. A number of genes potentially involved in rice blast resistance were identified from these studies. In a previous transcriptome study on the incompatible interaction mediated by *Pi33*, seven genes were up-regulated more than 1.5 fold and 13 were down-regulated more than 2 fold at 24 hpi [26]. Given a broad range of genes responsible for plant defense response [27], [28], the defense transcriptome at early stage of rice–*M. oryzae* interactions is far from complete and the underlying mechanisms remain largely unclear.

To better understand the defense mechanisms underlying rice early response to *M. oryzae*, we carried out a whole-genome transcriptional analysis on both compatible and incompatible rice–*M. oryzae* interactions at 24 hpi. Our results showed that the transcriptional profiles of the compatible and incompatible interactions were mostly similar, with higher transcriptional changes in the incompatible interactions. Functional analysis revealed that the genes involved in signaling, secondary metabolism, and those encoding WRKY transcription factors, were significantly enriched among up-regulated genes. Overexpression of one such *WRKY* gene, *OsWRKY47*, greatly enhanced rice resistance to *M. oryzae*. Gene set enrichment analysis further revealed different regulation patterns among functional categories, suggesting genes with different functions were subjected to distinct regulation programming. This transcriptome study provided a comprehensive overview of transcriptional reprogramming during rice early response to *M. oryzae*.

## Results

### Transcriptional Profiles of Rice Compatible and Incompatible Interactions with *M. oryzae* are Similar Overall

**Figure 1:**
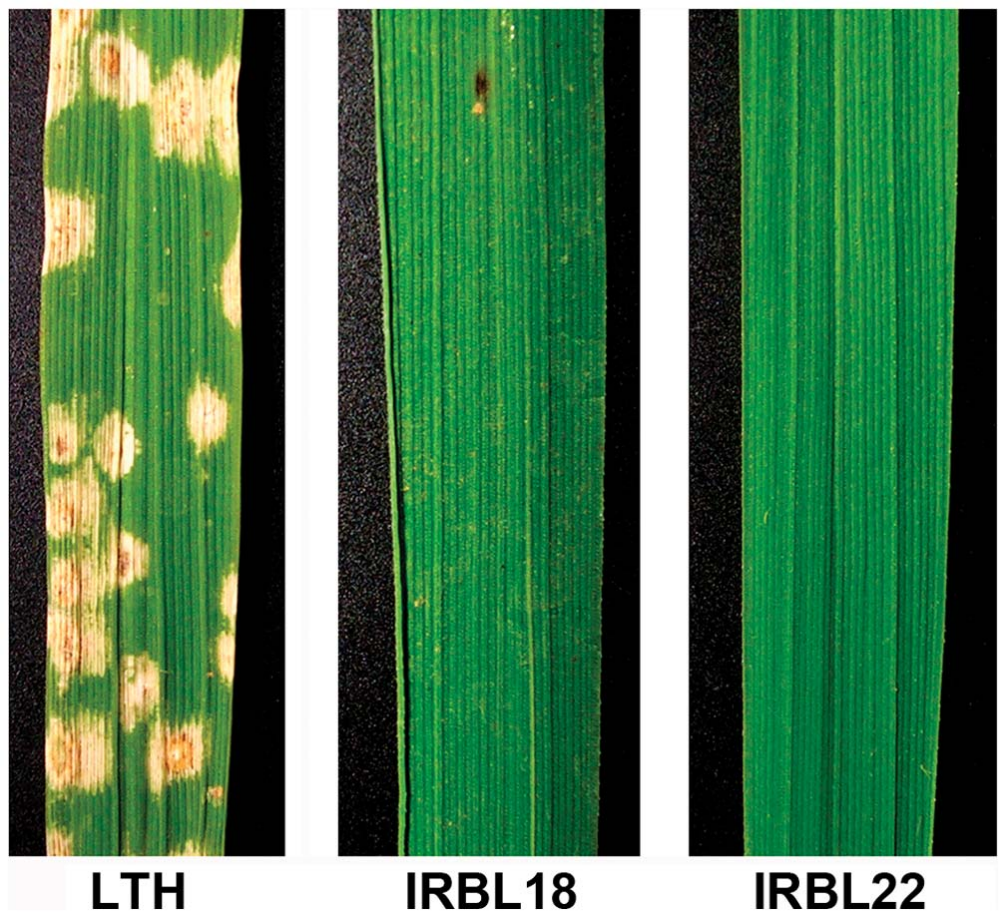
The disease symptoms on IRBL18, IRBL22 and LTH leaves at 7 days post-inoculation. The inoculation experiment was repeated three times (n = 10 seedlings) with similar results.

To investigate the defense mechanisms underlying rice early response to *M. oryzae*, we conducted a microarray-based transcriptome study on compatible and incompatible rice–*M. oryzae* interactions at 24 hpi. One susceptible rice cultivar, LTH, and two resistant near-isogenic lines (NILs), IRBL18 and IRBL22, were used in this study (Figure 1). In our previous study, IRBL18 (carrying *Pi1*) and IRBL22 (carrying *Pi9*) exhibited broad-spectrum resistance to more than 200 *M. oryzae* strains collected in Yunnan Province of China [29]. *Pi9* encodes a typical nucleotide binding and leucine rich repeat (NB-LRR) protein and confers resistance to another 21 blast strains from nine countries [30]. In this study, the incompatible interactions in IRBL18 and IRBL22 were used to investigate the R-mediated ETI response, while the compatible interaction in susceptible LTH represented the PTI response (Figure 1).

**Figure 2:**
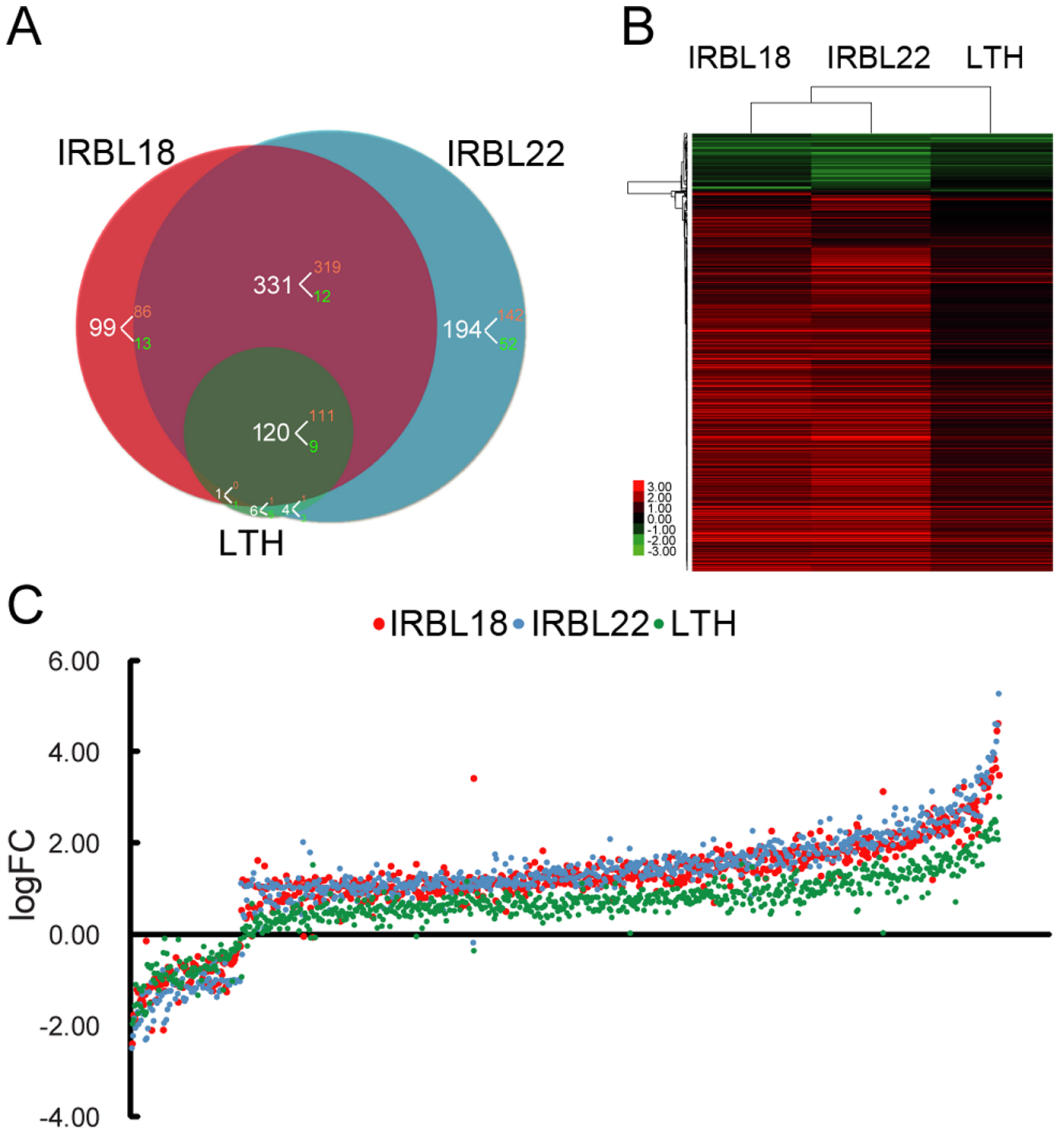
Differentially expressed genes (DEGs) identified from the transcriptome study. **A:** The venn diagram of DEGs in IRBL18, IRBL22 and LTH. The numbers of up- and down-regulated genes were shown in red and green, respectively. **B:** Hierarchical clustering of the log2-transformed fold changes. The scale bar displays log2-transformed fold changes. **C:** Scatter plot of log2-transformed fold changes.

For microarray analysis, four-week-old rice seedlings were inoculated with fungal spore suspension or mock-inoculated with spore-free water. Microarray analyses were performed using Affymetrix GeneChips with three independent biological replicates. We identified a total of 755 differentially expressed genes (DEGs; fold change ≥2; *P*<0.05; listed in Table S1) between the fungal- and mock-inoculated IRBL18, IRBL22 or LTH plants. Twenty genes were validated by quantitative RT-PCR (Table S2). Among the 755 DEGs, 551 were differentially regulated in IRBL18, 649 in IRBL22 and only 131 in LTH. The number of DEGs in either IRBL18 or IRBL22 was about four times more than that in LTH (Figure 2A), suggesting that the two incompatible interactions involved more expanded genome-wide transcriptional regulation than did the compatible interaction. We also found that the up-regulated genes greatly outnumbered the down-regulated ones in either rice–*M. oryzae* interaction, demonstrating that the majority of genes involved in rice early response to *M. oryzae* were positively regulated (Figure 2B). Interestingly, 451 DEGs were shared by IRBL18 and IRBL22, and 120 of them were also differentially regulated in the susceptible LTH plants (Figure 2A). This reflects some basal similarities between the transcriptomes in rice compatible and incompatible interactions with *M. oryzae*.

A hierarchical clustering analysis was conducted on the log2-transformed fold change values (logFCs) of 755 DEGs. The transcriptional profiles in IRBL18 and IRBL22 were similar with comparable amplitudes (Figure 2B), indicating that the *Pi1*- and *Pi9*-mediated pathogen recognitions triggered similar ETI responses in the transcriptome term. Interestingly, the transcriptional profile in LTH was also similar to those in IRBL18 and IRBL22, but the amplitudes of gene expression changes were globally lower. Differences in the transcriptional profiles between compatible and incompatible rice–*M. oryzae* interactions were mostly quantitative, especially in the up-regulated DEGs (Figure 2C). These results suggest that a common set of defense-responsive genes are involved in rice early defense response to *M. oryzae*, and that they are more prominently regulated in rice incompatible interactions.

### Transcriptional Reprogramming in Signaling and Metabolism during Rice Early Response to *M. oryzae*

As an important step towards understanding the molecular mechanisms underlying rice early response to *M. oryzae*, we functionally analyzed the 755 DEGs with their Gene Ontology (GO) annotations. The enrichment analysis showed that “kinase activity” was the most significantly enriched GO molecular function term among up-regulated genes in each rice line (*P = *5.61×10^−21^ in IRBL18, 2.38×10^−17^ in IRBL22, and 4.59×10^−4^ in LTH), indicating that a significant number of kinase genes were positively regulated during rice early response to *M. oryzae* (Table 1). Both “RNA binding” and “DNA binding” were depleted among the up-regulated genes in IRBL18 or IRBL22 (*P<*0.05), suggesting that most genes were not under positive transcriptional regulation. No significantly enriched or depleted GO terms were found in the down-regulated genes, partially because a small number of genes were suppressed.

**Table 1:** The enrichment analysis of GO in the up-regulated genes.

GO ID	GO term	Number in reference	IRBL18		IRBL22		LTH	
			count	*P*-value	count	*P*-value	count	*P*-value
total		17,752	516		573		113	
Over-represented								
GO:0016301	kinase activity	1,086	94	<<0.001	93	<<0.001	19	<0.001
GO:0030246	carbohydrate binding	108	12	<0.001	14	<0.001	3	0.158
GO:0019825	oxygen binding	121	12	0.001	11	0.007	3	0.184
GO:0005515	protein binding	2,130	82	0.018	95	0.003	21	0.158
GO:0005215	transporter activity	911	33	0.265	47	0.004	9	0.492
Under-represented								
GO:0003677	DNA binding	879	11	0.008	15	0.039	3	1.000
GO:0003723	RNA binding	504	1	<0.001	2	<0.001	0	1.000

*P*-values were corrected with the Benjamini-Hochberg’s method.

**Figure 3:**
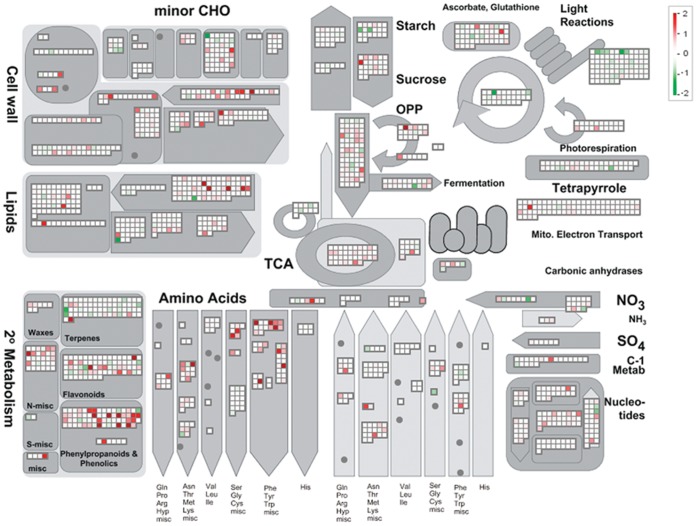
Overview of the transcriptional changes in IRBL18 at 24 hours post-inoculation. Genes significantly up- (red) or down-regulated (green) in fungal-inoculated leaf samples relative to mock-inoculated are illustrated. The scale bar displays log2-transformed fold changes.

We then performed a more detailed analysis using MapMan with a manually updated mapping file [31]. Among the 755 DEGs, 551 were assigned to 28 known functional categories (Figure 3). The statistical significances of their enrichments in each rice line were assessed with the 17,752 expressed genes as reference. In the resistant IRBL18 and IRBL22, the genes involved in “signaling”, “amino acid metabolism”, “secondary metabolism”, “cell wall synthesis” and “stress response” were significantly enriched in the up-regulated DEGs (*P<*0.05; Table 2), suggesting that the resistant rice plants went through a transcriptional reprogramming, both in signaling and metabolism, during their early responses to *M. oryzae*. However, in the susceptible LTH, only three categories, “signaling”, “amino acid metabolism” and “secondary metabolism”, were enriched, partially due to the limited transcriptional reprogramming in compatible interaction.

**Table 2:** The enrichment analysis of functional categories in the up-regulated genes.

Functional categories	Number in reference	IRBL18		IRBL22		LTH	
		count	*P*-value	count	*P*-value	count	*P*-value
Total	17,752	516		573		113	
Over-represented categories							
Signaling	1,275	105	<<0.001	107	<<0.001	19	0.011
Amino acid metabolism	229	19	<0.001	20	<0.001	6	0.045
Secondary metabolism	255	27	<0.001	27	<0.001	8	0.008
Cell wall synthesis	195	13	0.032	14	0.029	2	1.000
Stress response	481	32	<0.001	35	<0.001	5	0.830
Transport	721	30	0.163	42	0.001	9	0.357
Under-represented categories							
RNA regulation	1,903	27	<0.001	30	<0.001	8	1.000
DNA regulation	317	1	0.024	1	0.009	0	1.000
Protein metabolism	2,527	38	<0.001	42	<0.001	4	0.038

*P*-values were corrected with the Benjamini-Hochberg’s method.

Consistent with GO analysis results, the functional category “RNA regulation” was significantly depleted in the up-regulated DEGs of IRBL18 and IRBL22 (*P*<0.001; Table 2). However, within this significantly depleted category, the WRKY transcription factor family was the only significantly-enriched one among 14 tested transcription factor families (*P = *1.47×10^−6^ in IRBL18, 3.00×10^−5^ in IRBL22, and 4.33×10^−2^ in LTH), suggesting that these WRKYs were likely to play crucial roles in rice early response to *M. oryzae* (see below).

### Genes Encoding Signaling Components were Extensively Up-regulated

Functional analysis indicated that “signaling” was the most significantly enriched functional category among the up-regulated genes in IRBL18 and IRBL22 (*P* = 5.96×10^−21^ in IRBL18 and 2.33×10^−18^ in IRBL22) and the second most significantly enriched in LTH up-regulated genes (*P = *1.05×10^−2^; Table 2). Of the up-regulated DEGs, 20.3% encoded signaling components in IRBL18, 18.7% in IRBL22, and 16.8% in LTH, significantly more than 7.2% in the reference data set. Thus, a broad range of genes involved in signaling pathways were up-regulated during rice early response to *M. oryzae*.

**Figure 4:**
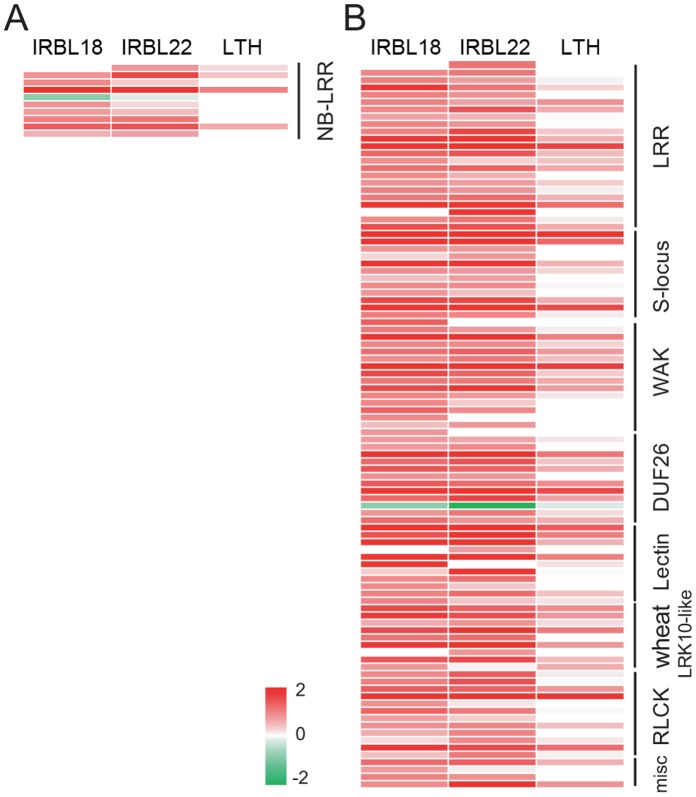
The expression patterns of receptor kinase and nucleotide binding and leucine rich repeat (*NB-LRR*) genes. **A:** The expression patterns of *NB-LRR* genes. **B:** The expression patterns of receptor kinase genes, with the family names shown on the right side. The scale bar displays log2-transformed fold changes.

Further analysis showed that within “signaling”, the sub-category “receptor kinase” was even more significantly enriched in the up-regulated genes (*P = *5.00×10^−35^ in IRBL18, 1.53×10^−31^ in IRBL22, and 2.33×10^−6^ in LTH). A total of 103 receptor kinase genes were up-regulated in either rice line, whereas only one was down-regulated (Figure 4B; Table S4). Moreover, this enriched sub-category contained several receptor kinase sub-families, including receptor-like kinases (RLKs) with various extracellular domains, receptor-like cytoplasmic kinases (RLCKs), and wall-associate kinases (WAKs), some of which were also significantly enriched in the up-regulated genes (Table 3). So many receptor kinase genes being up-regulated suggested that signal perception was extensively activated early in *M. oryzae* infection. In contrast to 103 up-regulated receptor kinases, only nine *NB-LRR* genes were up-regulated (Figure 4A), although NB-LRRs are usually responsible for specific recognition of pathogen Avr proteins to trigger the ETI response. The different expression patterns between receptor kinase and *NB-LRR* genes suggested that they are subjected to distinct transcriptional regulation procedures in rice early response to *M. oryzae*.

**Table 3:** The enrichment analysis of receptor kinases in the up-regulated genes.

Sub-family names	Number in reference	IRBL18		IRBL22		LTH	
		counts	*P*-value	counts	*P*-value	counts	*P*-value
total	17,752	516		573		113	
Leucine rich repeat I	12	3	0.031	3	0.036	0	1.000
Leucine rich repeat X	16	4	0.008	2	0.343	0	1.000
Leucine rich repeat VIII	34	5	0.022	5	0.029	1	0.830
Leucine rich repeat VIII-1	31	5	0.015	5	0.022	1	0.830
DUF 26	36	12	<0.001	10	<0.001	3	0.031
Legume-lectin	35	9	<0.001	9	<0.001	3	0.031
Wheat LRK10 like	21	7	<0.001	8	<0.001	3	0.010
S-locus glycoprotein like	62	10	<0.001	11	<0.001	2	0.488
Wall associated kinase	51	14	<0.001	12	<0.001	1	0.950
RLCK	113	7	0.207	10	0.027	2	0.827

*P*-values were corrected with the Benjamini-Hochberg’s method.

Other signaling component genes with known functions in rice resistance were also up-regulated during the early response to *M. oryzae*. For instance, *NH1* (*LOC_Os01g09800*), a positive regulator in rice blast resistance, was 2.5-, 3.5-, and 2.0-fold up-regulated in IRBL18, IRBL22, and LTH, respectively. Two MAP kinase genes (*LOC_Os03g17700* and *LOC_Os02g04230*) were also up-regulated, in which *LOC_Os03g17700* encodes an negative regulator, OsMPK5, in blast resistance [32]. Another five calmodulin-binding protein genes (*CBP*s) and two calcium/calmodulin-dependent protein kinase genes (*CDPK*s) were also up-regulated (Table S1), suggesting the calcium signaling was involved as well.

### Genes Involved in Both Shikimate and Phenylpropanoid Pathways were Up-regulated

**Figure 5:**
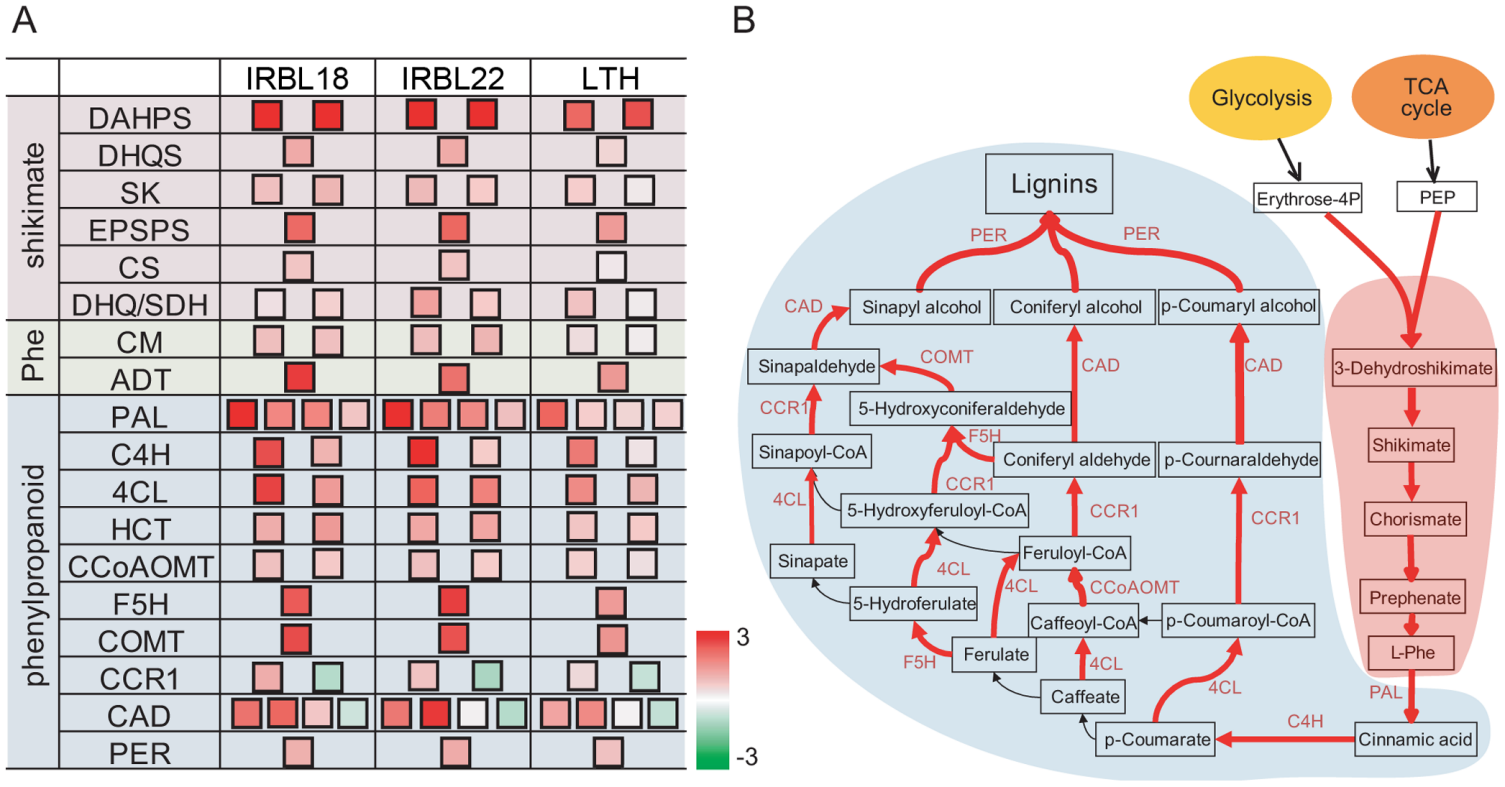
Transcriptional changes in shikimate and phenylpropanoid biosynthesis pathways. **A:** The expression patterns of genes in the shikimate and phenylpropanoid biosynthesis pathway. The scale bar displays log2-transformed fold changes. **B:** The metabolic view of shikimate and phenylpropanoid biosynthesis pathways. The steps catalyzed by enzymes encoded by up-regulated genes were shown in red. The shikimate and phenylalanine biosynthesis pathways were highlighted with pink background and the phenylpropanoid biosynthesis pathway was highlighted with light blue background. Gene abbreviations were listed in Table S5.

Our data showed that a significant number of genes involved in amino-acid and secondary metabolism were up-regulated during rice early response to *M. oryzae* (Table 2; Table S5). To better illustrate the transcriptional regulation of rice metabolism during this process, we integrated the up-regulated genes in these pathways into an overall metabolic view (Figure 5). Notably, dozens of genes involved in amino acid and secondary metabolism were up-regulated and formed a metabolic map consisting of the shikimate and phenylalanine biosynthesis pathways as well as the downstream phenylpropanoid biosynthesis pathway. In the shikimate biosynthesis pathway, six out of 14 expressed enzyme genes were up-regulated in either rice line, whose products catalyze five successive reactions. Following the transcriptional activation of shikimate biosynthesis pathway, the genes encoding chorismate mutase and prephenate dehydratase, catalyzing chorismate to form phenylalanine, were also up-regulated. In the downstream, the phenylpropanoid biosynthesis pathway was transcriptionally activated, *i.e.*, 19 genes out of 80 various enzyme genes were up-regulated. Like the shikimate pathway, the enzymes encoded by these 19 genes catalyze a series of eight reaction steps in phenylpropanoid pathway. These results showed that both shikimate and phenylpropanoid pathways were under an expanded transcriptional activation during rice early response to *M. oryzae*, indicating the transcriptional reprogramming occurred in metabolic pathways.

Moreover, eight of 34 enzyme genes involved in jasmonic acid biosynthesis were up-regulated in either rice line (Table S6), suggesting that the jasmonic acid biosynthesis pathway was activated during rice early response to *M. oryzae*. In addition to the genes involved in established metabolic pathways, other individual enzyme gene families, including cytochrome P450 s and peroxidases, were also significantly enriched in the up-regulated DEGs. In plant defense response, cytochrome P450 s and peroxidases are often involved in modifying a plethora of anti-pathogen proteins. The enrichment of these genes suggested that these enzymes could function as active players in the defense response against rice blast fungus.

### Transcriptional Differences Between ETI and PTI Varied Among Functional Categories

**Figure 6:**
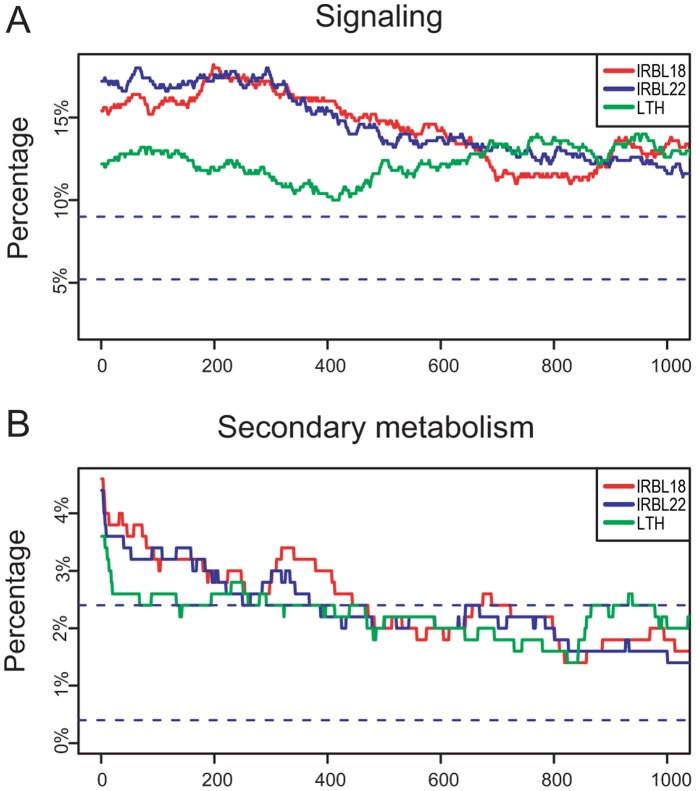
The sliding window analysis of genes involved in signaling (A) and secondary metabolism (B). The solid lines represent the percentages of certain functional category in a 500-gene window in IRBL18 (red), IRBL22 (blue) and LTH (green). The blue dotted lines represent the 95% confidence levels.

In the functional analyses above, we found that differences in the transcriptional profiles between compatible and incompatible rice–*M. oryzae* interactions were mostly quantitative (Figure 2). This result raised an interesting question, *i.e.*, whether these quantitative differences in transcriptional profiles were of the same scale across functional categories. To address this question, we carried out two complementary analyses. First, we compared the enrichment of functional categories among rice lines. All 17,752 expressed genes were sorted by descending their *t*-statistic values. The percentage of a given functional category in a 500-gene sliding window was calculated and plotted along the gene list. For the “signaling” category, the lines representing the percentages of signaling components were above the upper threshold (Figure 6A), demonstrating that “signaling” was significantly enriched among the up-regulated genes, consistent with the enrichment analysis above (Table 2). However, among the nearly one thousand most-induced genes, signaling components comprised around 15% in IRBL18 and IRBL22 and only 12% in LTH, reflecting a 3% difference in this functional category between incompatible and compatible interactions (on the left in Figure 6A). This result suggested that the genes encoding signaling components were not only more prominently (Figure 4B) but also more extensively up-regulated in the incompatible interactions than in the compatible interaction.

The pattern was different in “secondary metabolism” (Figure 6B). For each rice line, the percentage of genes involved in secondary mechanism was high within the most up-regulated genes, suggesting that these genes tended to be intensively up-regulated during rice early response to *M. oryzae*. The difference between IRBL18 or IRBL22 and LTH was about 1% within the most up-regulated genes, also demonstrating that more genes involved in secondary metabolism were up-regulated in the two incompatible rice–*M. oryzae* interactions. By this method, we found that there were more genes in “amino acid metabolism”, “cell wall synthesis”, “stress response”, and “transporter” up-regulated in the resistant IRBL18 or IRBL22 than in the susceptible LTH (Figure S1). These results demonstrated that the transcriptional regulations in various functional categories differed not only in global patterns but also between compatible and incompatible interactions.

To quantitatively analyze differences within functional categories between interactions, we performed a permutation-based Gene Set Enrichment Analysis (GSEA) [33]. The null hypothesis that genes in a given category had equal transcriptional changes in the compatible and incompatible interactions (see Materials and Methods) was tested. “Secondary metabolism” and “amino acid metabolism” exhibited the most differences between IRBL18 or IRBL22 and LTH (*P*<0.05; Table 4). The “signaling” and “stress response” differed to a lesser degree between the incompatible and compatible interactions (*P*<0.1). More importantly, *WRKY* genes were also more prominently regulated in IRBL18 and IRBL22 than in LTH in a less degree (*P = *0.0664 in IRBL18 vs. LTH, 0.0815 in IRBL22 vs. LTH, 0.410 in IRBL18 vs. IRBL22). Given their small number (45), the crucial roles of these WRKYs in rice blast resistance were apparent according to the expression patterns demonstrated here.

**Table 4:** The result of Gene-set enrichment analysis on functional categories.

Functional categories	IRBL18 vs LTH*P-*value	IRBL22 vsLTH*P-*value	IRBL18 vs IRBL22*P-*value
Secondary metabolism	0.0079	0.0242	0.1785
Amino acid metabolism	0.0255	0.0292	0.4445
Stress response	0.0314	0.0815	0.2068
Lipid metabolism	0.0429	0.1288	0.1562
Signaling	0.0574	0.0962	0.3255
Protein metabolism	0.0583	0.1571	0.1804
Hormone metabolism	0.07	0.3105	0.0917
Transporter	0.0746	0.1392	0.255
DNA regulation	0.0972	0.2947	0.135
Cell wall modification	0.1084	0.1221	0.5054
RNA regulation	0.1142	0.3158	0.1267
Nucleotide metabolism	0.1197	0.1657	0.3717
Redox regulation	0.1352	0.3261	0.1332
Development	0.3478	0.4245	0.3777
Photosynthesis	0.4271	0.4195	0.5273

### WRKY Transcription Factor Genes play Important Roles in Rice Blast Resistance

**Figure 7:**
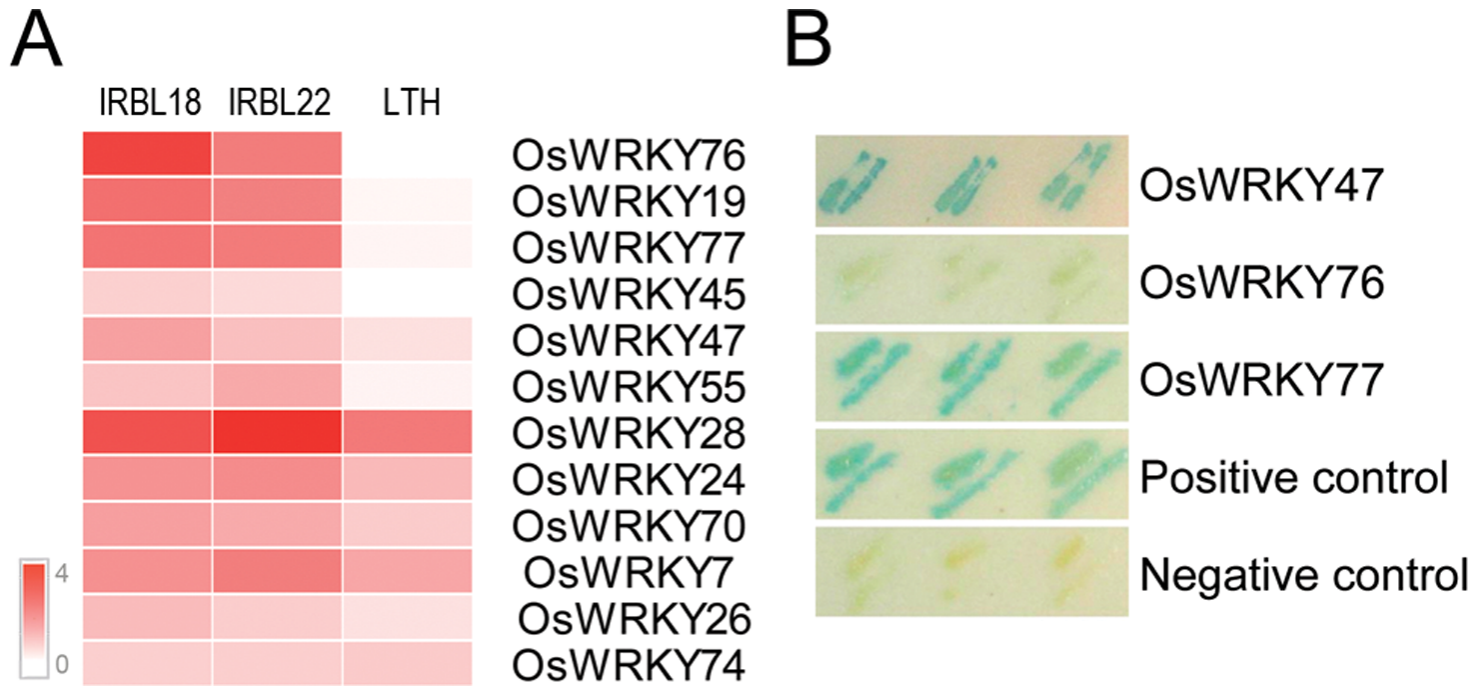
The expression patterns of OsWRKYs (A) and the trans-activation activities of selected OsWRKYs (B). The scale bar in (A) displays log2-transformed fold changes.

Among the 45 *WRKY* genes expressed during rice early response to *M. oryzae*, eleven were up-regulated in IRBL18, ten in IRBL22, and only three in LTH (Table 5). *WRKY* transcription factor genes were significantly enriched in the up-regulated DEGs in each rice line (*P*<0.001 in IRBL18 and IRBL22, and *P* = 0.0433 in LTH). Interestingly, these *WRKY*s showed different expression patterns between compatible and incompatible interactions (Figure 7A). For instance, *OsWRKY76* was significantly induced in both resistant rice lines, with 8.8-fold up-regulation in IRBL18 and 4.6-fold in IRBL22, whereas no change occurred in LTH. *OsWRKY47* was up-regulated 3.3-fold in IRBL18, 2.4-fold in IRBL22, and only 1.8-fold in LTH (Table S7; also validated in Table S2). Among these WRKYs, OsWRKY45 [19] and OsWRKY55 (designated as OsWRKY31 in [20]) have been demonstrated to play positive roles in rice–*M. oryzae* interactions, further supporting our microarray results.

**Table 5:** The summary of differentially regulated transcription factor genes.

Family names	Number in reference	IRBL18		IRBL22		LTH	
		up	down	up	down	up	down
total	17,752	516	35	573	76	113	18
WRKY	46	11	0	10	0	3	0
MYB	51	4	0	3	0	2	0
GARP	31	2	0	2	0	0	0
HSF	18	2	0	2	0	0	0
NAC	55	2	0	2	1	1	0
GRAS	23	1	0	1	0	0	0
bHLH	74	1	1	2	1	1	0
DOF	16	1	0	1	0	0	0
AP2/EREBP	69	0	0	4	1	0	0
bZIP	59	0	1	0	2	0	1
Tify	13	0	0	1	0	0	0
GATA	14	0	0	1	0	0	0

**Figure 8:**
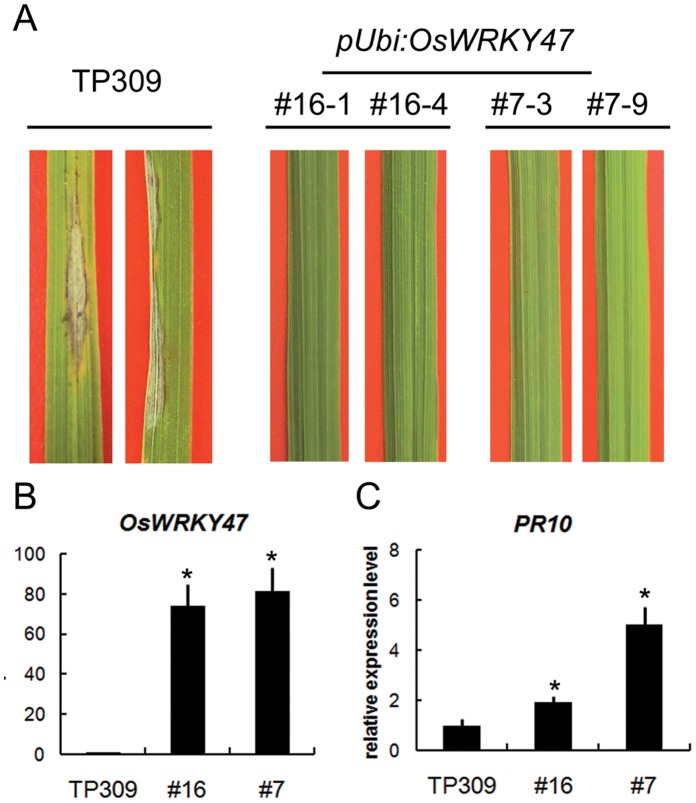
Overexpression of *OsWRKY47* enhanced rice resistance to *Magnaporthe oryzae* strain 96-4-1a. **A:** The disease symptoms on TP309, *pUbi:OsWRKY47*#16 and #7 transgenic plants at 7 days post-inoculation. The inoculation experiments were repeated three time (n = 15 seedlings) with similar results. **B,** **C:** The expression levels of *OsWRKY47* and *PR10* in TP309 and *pUbi:OsWRKY47* transgenic plants determined by quantitative RT-PCR. The expression levels were standardized to *OsUBQ* and the value in TP309 was set at 1.0. Error bars in (B) and (C) represent the SD from three biological replicates, each containing at least 10 seedlings. The expression levels that differ significantly (*P*<0.05) from those in TP309 were marked with asterisks.

To further verify the biological roles of WRKY transcription factors in rice blast resistance, we select three *OsWRKY*s with different expression patterns, including *OsWRKY47*, *OsWRKY76* and *OsWRKY77*, for further functional analyses. The yeast assay showed that OsWRKY47 and OsWRKY77 exhibited trans-activation activity, while OsWRKY76 did not (Figure 7B), suggesting these up-regulated *OsWKRY*s encoded transcription regulators with different functions. We then overexpressed these three *OsWRKY*s in rice cultivar Taipei 309 (TP309). Interestingly, the transgenic plants overexpressing *OsWRKY76* or *OsWRKY77* were severely compromised in their viability (data not shown). Twenty-two independent *OsWRKY47* overexpression transgenic lines were obtained and two lines (*pUbi:OsWRKY47*#7 and *pUbi:OsWRKY47*#16) with constitutively-high expression levels of *OsWRKY47* were selected for rice blast resistance assay (Figure 8B). Seven days after inoculation with *M. oryzae* strain 96-4-1a, typical lesions with grey centers occurred and spread on the leaves of wild-type rice plants, while no visible lesions were observed on the leaves of two *pUbi:OsWRKY47* transgenic lines, indicating that the transgenic plants exhibited greatly enhanced blast resistance (Figure 8A; Table S8). Consistent with the enhanced blast resistance, the expression levels of the marker gene *Pathogenesis-related 10* (*PR10*) were significantly higher in the transgenic plants than in the wild-type TP309 (Figure 8C). These results demonstrated that OsWRKY47 played an important role in rice blast resistance.

## Discussion

After decades of study of rice–*M. oryzae* interactions, a number of blast resistance genes have been mapped and identified, and many have been successfully applied in the field. However, because of blast population diversity and genetic instability, many newly released rice cultivars have a short lives of only 2–3 years [6], [12], increasing the urgency of studying the molecular mechanisms underlying rice–*M. oryzae* interactions. Here, we carried out a transcriptome study on rice compatible and incompatible interaction with *M. oryzae* at a critical early stage of 24 hpi. A total of 755 differentially-regulated genes were identified from *M. oryzae*-challenged rice plants, including genes encoding receptors, signaling transducers, transcription factors, and enzymes involved in metabolic pathways. Our study provided an overview of transcriptional reprogramming during rice early defense response to blast fungus.

Rice resistance against blast fungus obeys the “gene-for-gene” hypothesis [8]. In this study, we used two resistant NILs, IRBL18 and IRBL22, to investigate rice early response in incompatible interactions with *M. oryzae*, and used their susceptible parent LTH to investigate a compatible interaction. By comparing these three transcriptomes, we found that the *Pi1-* and *Pi9-*mediated ETI responses in IRBL18 and IRBL22 shared similar transcriptional profiles to the PTI response in LTH, with stronger transcriptional changes during the ETI responses (Figure 2). These patterns resembled those in the *Arabidopsis thaliana*–*Pseudomonas syringae* interaction, in which the transcriptional profiles of compatible and incompatible *A. thaliana*–*P. syringae* interactions at the same time points were similar and the differences in profiles were mostly quantitative [4]. In another well-studied barley–powdery mildew interaction, similar transcriptional regulation patterns were also found between compatible and incompatible interactions at an early stage [28], [34]. In the innate immune system, plants detect the potential pathogens via PRRs and R proteins, activating PTI and ETI, respectively. Although triggered by different mechanisms, the transcriptional signatures in PTI and ETI are largely similar, suggesting that their responses are the same overall but differ in magnitude [2]–[4]. Our results indicate that the rice–*M. oryzae* interaction shows a quantitative nature similar to other plant–pathogen interactions.

During the functional analysis, we noticed that some functional categories, such as “signaling”, comprised a greater percentage of DEGs in IRBL18 and IRBL22 than in LTH. This raised the interesting question of whether the quantitative differences were of the same scale across the whole defense transcriptome. As functional enrichment analyses could be only conducted with a certain gene list with a given cutoff, we carried out two complementary analyses with all expressed genes. Assuming that all transcriptional changes were proportionally higher in the incompatible interactions, the genes in any given category should have a similar distribution when ranking according to their significance of transcriptional changes. Interestingly, this was not the case here. The percentages of enriched categories identified in functional analyses, including “signaling”, “stress response”, “secondary metabolism” and “amino acid metabolism”, in the most-induced genes were higher in the resistant IRBL18 and IRBL22 plants than in the susceptible LTH plants, and the differences in their transcriptional changes were significant to different extents (Table 4). These results suggested that the genes in these categories were under broader (Figure 6; Figure S1) as well as stronger (Figure 2) regulation in ETI than in PTI. Moreover, different functional categories seemed to exhibit different regulation patterns. For instance, the genes involved in signaling were broadly up-regulated among the first one thousand genes, while those involved in secondary mechanism were mainly enriched in only a few hundred (Figure 6B). Given that “secondary mechanism” was the most significantly different functional category between ETI and PTI, and enriched in the most up-regulated genes, it indicated that the secondary metabolism genes were highly induced during rice early response to *M. oryzae* (Table 4). The extensive regulation in signaling and intensive regulation in metabolism suggested that different functional categories within rice immune system were under distinct transcriptional regulation mechanisms, which might reflect the amplification of defense signals via signaling cascades.

Plant immunity is governed by sophisticated systems involving perception receptors, signaling mediators, transcriptional regulators, and a variety of anti-pathogen proteins [2], [3], [13], [27]. Among the functional categories enriched among up-regulated genes, we found that genes involved in signaling were the most significantly enriched in IRBL18 and IRBL22, in which a significant number (103) of receptor kinase genes were up-regulated (Figure 4; Table S4). Receptor kinases, which comprise one of the largest plant gene families, are often responsible for perceiving internal and external signals [35]–[37]. In *Arabidopsis*, FLS2 functions as a PRR receptor to recognize bacterial flagellin, leading to a PTI response [38]–[40]. OsFLS2, the rice homolog of FLS2, has been also identified as a flagellin perception receptor [41], and its encoding gene was up-regulated 1.9- and 1.7-fold in IRBL18 and IRBL22, respectively. *OsWAK25* (*LOC_Os03g12470*) encodes a receptor with a positive role in resistance to the bacterial pathogen *Xanthomonas oryzae* pv. *oryzae* [42] and was also up-regulated during rice early response to *M. oryzae*. The extensive up-regulation of receptor kinases genes, including those responding to other pathogens, suggested that rice plants challenged by *M. oryzae* might activate a broad range of perception receptors to prepare for further potential infections by pathogens, such as *X. oryzae*.

The WRKY transcription factor family was one of the most notably enriched gene families identified in this study, because it was the only enriched transcription factor family within the significantly depleted “RNA regulation” category (Table 2). Our results indicated that WRKY transcription factors play important roles in rice blast resistance, as was previously demonstrated by genetic approaches. For example, overexpressing *OsWRKY45*, *OsWRKY55*, and *OsWRKY47* enhanced rice blast resistance, indicating their positive roles in rice defense response to *M. oryzae* [19], [20]; this study). Knockdown of *OsWRKY28* led to an increase in blast resistance, indicating its negative role [43]. Moreover, Xie *et al.* demonstrated that OsWRKY72 and OsWRKY77 played synergistic roles with abscisic acid (ABA) in aleurone cells, and OsWRKY24 and OsWRKY45 repressed ABA induction of downstream genes, reflecting OsWRKYs involvement in plant development [44]. In *Arabidopsis*, WRKY transcription factors with both positive and negative regulatory roles form a complex transcriptional network to modulate plant defense responses [45]. Therefore, the up-regulation of these *OsWRKY* genes, whose products possess distinct trans-activation activity and biological roles, suggested that a complex network of rice WRKY transcription factors could also be activated to fine-tune the early responses to *M. oryzae*.

In the downstream of defense signaling pathways, many defense-related genes are transcriptionally regulated to actively respond to invasive pathogens [2], [3], [27]. The phenylpropanoid biosynthesis pathway is of great importance in this process, because phenylpropanoids are important antimicrobial compounds and lignification plays an important role in fungal resistance [46], [47]. Down-regulation of phenylpropanoid biosynthesis genes compromises plant resistance to fungal pathogens [46], [48]–[50]. These biosynthesis genes are under coordinated transcriptional regulation, particularly by MYB transcription factors [51]–[53]. In this transcriptome study, we found that many enzyme genes involved in the phenylpropanoid biosynthesis pathway were up-regulated, as were those in the upstream shikimate and phenylalanine biosynthesis pathways, reflecting a transcriptional reprogramming in metabolism during rice early response to *M. oryzae*. This transcriptional reprogramming was correlated with massive metabolic changes during rice–*M. oryzae* interactions, in which dozens of metabolites accumulated in rice leaves within 48 h of challenge by *M. oryzae* [54]. Moreover, nearly 40% of the “explanatory” *m/z* signals were predicted to derive from phenylpropanoid metabolites, highlighting our finding of transcriptional reprogramming in plant metabolism prior to an active metabolic response.

In summary, we demonstrated that a transcriptional reprogramming in signaling and metabolism happened during rice early response to *M. oryzae*. By comparing the transcriptomes in compatible and incompatible rice–*M. oryzae* interactions, we revealed that the defense response to rice blast fungus was quantitative in nature, with distinct transcriptional regulation mechanisms in various functional categories. This study enhanced our understanding of the complex network of transcriptional regulation during rice early response to the blast fungus.

## Materials and Methods

### Plant Material and Growth Conditions

*Oryza sativa* L. ssp *japonica* cultivar LTH and two near isogenic lines with LTH background, IRBL18 and IRBL22 bred by International Rice Research Institute (IRRI), were used in this study. All rice seedlings were grown in a greenhouse at Yunnan Agricultural University from April to May in the growing season. Seeds were surface-sterilized, washed repeatedly and soaked in water for germination. Five days later, 30 well-germinated seeds of each rice line were planted in 35×25×8.5-cm trays filled with sterile soil. The trays were put in water tanks and watered every three days. Twenty grams of nitrogen equivalent were fertilized in each tray two weeks before inoculation. The same growth conditions were applied to both fungal- and mock-inoculated groups of rice seedlings.

### Fungal Inoculation and RNA Preparation

The rice blast fungus (*Magnaporthe oryzae*) strain CH63 was cultured as previously [29], and conidia were harvested by rinsing the cultural plates with sterile distilled water and filtering through two layers of gauze. For the inoculation group, four-week-old rice seedlings were inoculated by spraying a conidial suspension of 1×10^5^ conidia mL^−1^ with 0.02% Tween-20. For the control group, sterile water with 0.02% Tween-20 was sprayed instead. The fungal- and mock-inoculated rice seedlings were kept in dark inoculation chambers with 100% humidity at 26°C.

At 24 hours after the inoculation, the fully-expanded third and fourth leaves of 20 rice seedlings from each rice line were harvested, pooled together and immediately frozen in liquid nitrogen. The remaining ten seedlings were left for disease assay at 7 days post-inoculation (dpi). Total RNA was extracted from leaf samples with TRIzol agent (Invitrogen) and purified with RNeasy kit (Qiagen). The quality of RNA was assessed by determining the A260/A280 ratio of RNA and by gel electrophoresis. The whole experiment was repeated three times with two-day intervals, resulting in three independent biological replicates of RNA samples.

### Microarray Analysis

Affymetrix GeneChip Rice Genome Arrays (Affymetrix, Santa Clara, USA) were used in this transcriptome study. RNA quality assessment, RNA labeling and microarray hybridization were performed at Capitalbio Ltd. (Beijing, China) according to the manufacturer’s instructions. After hybridization, the Affymetrix GeneChip Scanner 3000 was used for microarray scan and the Affymetrix GeneChip Operating Software Version 1.0 was used for image analysis and data extraction.

For microarray analysis, we used a series of R/Bioconductor packages (http://www.bioconductor.org) [55]. Briefly, the CEL files were imported into R environment and the robust multi-array average (rma) methodology, as implemented in the affy package, was used for microarray normalization [56], [57]. The Pearson’s correlation coefficiencies of log2-transformed expression values between replicates ranged from 0.985 to 0.995, indicating high consistency between biological replicates (TableS9). Following normalization, a non-specific filtering step was carried out. The probe sets called “Present”, which were determined by the mas5 algorithm, in at least two among three replicates in at least one rice sample were regarded as expressed and included in further analyses. Bacterial control probe sets and ambiguous probe sets that match no gene or multiple genes annotated in MSU Rice Genome Annotation Project database release 7.0 (http://rice.plantbiology.msu.edu/; [58]) were also removed, resulting in a final 21,593-data set representing 17,752 expressed rice genes (see below).

Subsequently, the limma package was used to identify differentially expressed probe sets between the fungal-inoculated samples with mock-inoculated ones [59]. The resulting *P*-values were adjusted by Benjamini and Hochberg’s method to control the false discovery rates [60]. A cutoff of *P*-value of 0.05 and fold change of two was used as the criterion for significantly differentially expressed probe sets. Hierarchical clustering was performed using the Cluster program (version 3.0; http://www.falw.vu/~huik/cluster.htm) with the Pearson’s correlation similarity metric and the average linkage clustering method, and illustrated by the TreeView program [61]. The raw data and processed results were submitted to the National Center for Biotechnology Information (NCBI) Gene Expression Omnibus (GEO) database under accession number GSE41798.

### Gene Annotation and Functional Analysis

Due to the great changes in rice genome annotation after the release of Affymetrix GeneChip Rice Genome Array, we updated the probe sets annotations in the first place. Probe sequences were aligned against the latest version 7.0 of MSU-annotated gene models using BLASTN program [62]. The probe sets with more than half of its entire set of probes perfectly matched with one transcript were considered, as previously described [63]. The probe sets matching no gene or multiple genes were discarded as ambiguous probe sets. By this means, we re-annotated a total of 40,539 probe sets, representing 33,814 rice genes. For functional analysis, GOSlim assigned to these genes were retrieved from the MSU database [58] and used for an enrichment analysis based on the hypergeometric distribution, with *P-*values adjusted by the Benjamini-Hochberg’s method [60]. As rice GO classification was too general, we therefore carried out a more detailed analysis using the MapMan program (version 3.5.0) [31]. The mapping file, which assigned genes into functional categories, was also updated to the latest MSU rice genome annotation version 7.0, and integrated with annotations from additional databases: PlantTFDB [64] and plnTFDB [65] for rice transcription factors; rice kinase database [37] for rice kinases, and rice gene families information from MSU database. Among the 17,752 expressed genes, 10,617 genes were assigned to known functional categories. The statistical significances of the enrichment or depletion in functional categories were tested using a hypergeometric test, with the *P-*values adjusted by the Benjamini-Hochberg’s method [60].

### Gene Set Enrichment Analysis

To compare the transcriptional changes between the compatible and incompatible interactions, two complementary analyses were performed using all 17,752 expressed genes. Firstly, we carried out a sliding-window analysis. For each rice line, all expressed genes were sorted according to their *t*-statistic values, provided by the Bayesian models fitted in the limma package, in a decreasing order. The top of the sorted gene list included the most up-regulated DEGs and the bottom included the most down-regulated. The percentage of the genes in a given MapMan functional category within a 500-gene sliding-window was calculated and plotted along their ranks.

Secondly, we performed a gene-set enrichment analysis to quantitatively assess the differences in transcriptional changes between compatible and incompatible interactions [33]. The null hypothesis is that the difference in transcriptional changes between compatible and incompatible interactions was equal to zero, *i.e.*, (I_T_−I_M_)−(C_T_−C_M_) = 0, where I_T_, I_M_ represent the log2-transformed gene expression values in fungal- and mock-inoculated incompatible rice line; C_T_, C_M_ represent those in fungal- and mock-inoculated compatible rice line. As comparing the transcriptional changes between two groups, we used the Bayesian moderate *t*-statistic *t_n_*, implemented by the limma package, and the statistic *z_N_* for a given functional category containing *N* genes, in which 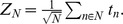 For each functional category, the observed *z_N_* statistic was calculated and the reference distribution of *z_N_* was generated with a ten thousand times of permutation of sample labels. The *P-*values were therefore calculated by comparing the observed *z_N_* statistic with its reference distribution.

### Quantitative RT-PCR

RNA samples used for the quantitative RT-PCR assay were the same as those for microarray hybridization. RNA treatment and cDNA synthesis was performed as previously described [66]. The quantitative RT-PCR was performed using the SYBR Green real-time PCR Master Mix (TOYOBO) on an Option 2 Continuous Fluorescence Detector (Bio-Rad), as previously described [67]. For each pair of primers, the amplification efficiency was assessed using LinRegPCR software [68] and the product specificity was examined by the melting curve and gel electrophoresis. Three independent biological replicates were performed for each sample. Cycling conditions were 1 min at 95°C, followed by 40 cycles of 30 sec at 95°C, 20 sec at 55°C, 20 sec at 72°C and plate read at an optimal temperature. *OsUBQ* was used as the internal control, and the 2^−ΔΔ*C*T^ method was used to calculate relative expression levels [69]. Primer sequences for the quantitative RT-PCR were listed in Table S3.

### Generation of Transgenic Rice Plants

The coding sequence (CDS) of *OsWRKY47* was amplified from rice cDNA by RT-PCR using primers (5'-ATG GCG TCT CCT GAT GGT GG-3', 5'-TTA AGG ATC GAA GCC AAA CA-3'). The PCR products were then cloned into pBluescript vector and confirmed by sequencing, resulting in the pBS-WRKY47 construct. To drive the constitutive expression of *OsWRKY47*, a maize *Ubiquitin* promoter was released by digesting pAHC25 [70] with *Hind* III/*BamH* I and ligated into the same restriction site of pBluescript vector, resulting the pBS-pUbq construct. Subsequently, pBS-WRKY47 was digested by *Spe* I/*Kpn* I, and the fragments including *OsWRKY47* CDS were ligated with *Hind* III/*Kpn* I digested pWM101 vector and the maize *Ubiquitin* promoter released from *Hind* III/*Spe* I digested pBS-pUbq, resulting in the pWM-WRKY47 overexpression construct. To generate transgenic plants for blast disease assay, we transformed the pWM-WRKY47 construct into the susceptible LTH cultivar using an *Agrobacterium*-mediated method [71]. However, the transformation efficiency for LTH was too low to obtain transformants. Therefore, we transformed the construct into another broadly-used blast-susceptible rice cultivar TP309. The pWM-WRKY47 construct was introduced into *A. tumefaciens* strain EHA105 and then transformed into TP309 calli, generating 22 independent transgenic lines with hygromycin resistance. T1-generation transgenic seedlings were screened for a possible single T-DNA insertion according to a 3∶1 (hygromycin-resistant/hygromycin-sensitive) segregation ratio. Homozygous T2-generation transgenic plants were used for blast disease assay. As described above, four-week-old wild-type TP309 and *pUbi:OsWRKY47* transgenic rice plants were inoculated with *M. oryzae* strain 96-4-1a and the disease symptom were assessed at 7 dpi,. The expression levels of *OsWRKY47* and *PR10* were examined by quantitative RT-PCR (primers listed in Table S3).

### Trans-activation Activity Assay

The CDS of *OsWRKY*s were cloned into pYF503 vector using primers listed below: OsWRKY47-F 5'-ATG GCG TCT CCT GAT GGT G-3' and OsWRKY47-R 5'-TTA AGG ATC GAA GCC AAA CA-3'; OsWRKY76-F 5'-ATG GAC GCG GCG TGG CGC-3' and OsWRKY76-R 5'-GAA TTC GGG CAG CTT CTG GAG G-3'; OsWRKY77-F 5'-ATG TCG TCG CTG TAC CCG TC-3' and OsWRKY77-R 5'-GTC AAG GAA GCA GCA GCG AG-3'. Trans-activation activity assay was conducted as previously described [72].

## Supporting Information

Figure S1 **The 500-sliding window analysis of the genes involved in amino acid metabolism (a), cell wall metabolism (b), stress response (c), transporter (d), lipid metabolism (e) and RNA metabolism (f).** The solid lines represent the percentages of certain functional category in a 500-sliding window in IRBL18 (red), IRBL22 (blue) and LTH (green). The blue dotted lines represent the 95% significance levels of greater or smaller than the reference.
(TIF)

Table S1 **List of 755 differentially regulated genes during rice early responses to** ***Magnaporthe oryzae*** **.**
(XLS)

Table S2 **List of quantitative RT-PCR results of selected genes.**
(XLS)

Table S3 **List of primers for quantitative RT-PCR.**
(XLS)

Table S4 **List of differentially regulated receptor kinase genes and nucleotide binding and leucine rich repeat (** ***NB-LRR*** **) genes.**
(XLS)

Table S5 **List of enzyme genes illustrated in** **Figure 5**.
(XLS)

Table S6 **List of differentially regulated genes involved in jasmonic acid biosynthesis pathway.**
(XLS)

Table S7 **List of differentially regulated transcription factor genes.**
(XLS)

Table S8 **The result of rice blast disease assay on** ***pUbi:OsWRKY47*** **transgenic plants.**
(XLS)

Table S9 **Pearson’s correlation efficiencies between biological replicates.**
(XLS)
