# Supplementary figures and images for: Transcriptional Profiling of Rice Early Response to Magnaporthe oryzae Identified OsWRKYs as Important Regulators in Rice Blast Resistance
Source: PLoS One. 2013 Mar 27;8(3):e59720. doi: 10.1371/journal.pone.0059720 (PMC3609760; doi:10.1371/journal.pone.0059720)

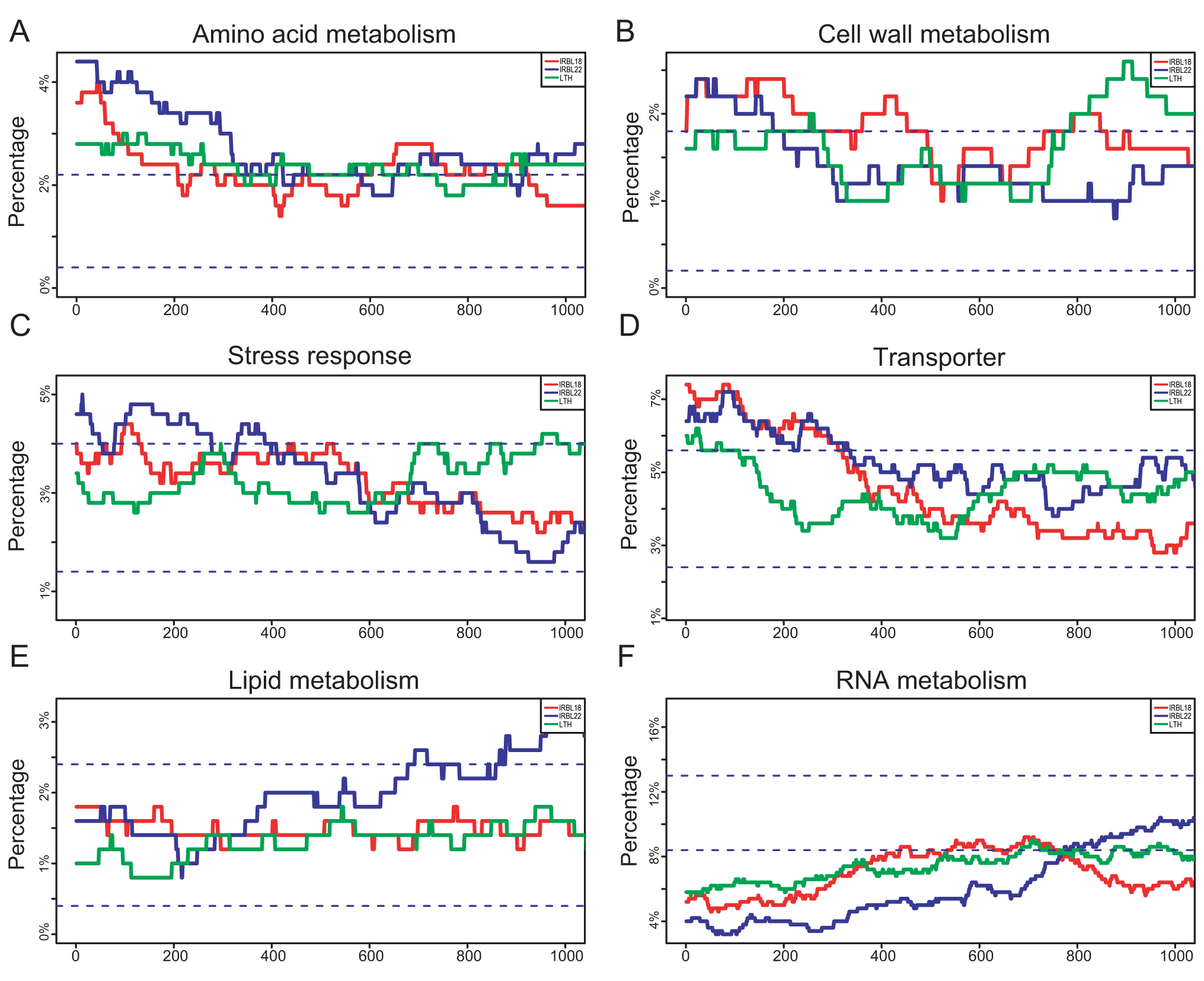

Supplement: Figure S1 — The 500-sliding window analysis of the genes involved in amino acid metabolism (a), cell wall metabolism (b), stress response (c), transporter (d), lipid metabolism (e) and RNA metabolism (f). The solid lines represent the percentages of certain functional category in a 500-sliding window in IRBL18 (red), IRBL22 (blue) and LTH (green). The blue dotted lines represent the 95% significance levels of greater or smaller than the reference. (TIF) [file pone.0059720.s001.tif]
